# Bacterial Superinfections After SARS-CoV-2 Pneumonia: Antimicrobial Resistance Patterns, Impact on Inflammatory Profiles, Severity Scores, and Clinical Outcomes

**DOI:** 10.3390/diseases13050145

**Published:** 2025-05-09

**Authors:** Petrinela Daliu, Iulia Bogdan, Ovidiu Rosca, Alexandra Laura Aelenei, Ioan Sîrbu, Mihai Calin Bica, Monica Licker, Elena Hogea, Delia Muntean

**Affiliations:** 1 Doctoral School, “Victor Babes” University of Medicine and Pharmacy Timisoara, Eftimie Murgu Square 2, 300041 Timisoara, Romania; petrinela.daliu@umft.ro; 2 Methodological and Infectious Diseases Research Center, Department of Infectious Diseases, “Victor Babes” University of Medicine and Pharmacy Timisoara, Eftimie Murgu Square 2, 300041 Timisoara, Romania; iulia-georgiana.bogdan@umft.ro (I.B.); ovidiu.rosca@umft.ro (O.R.); alexandra.aelenei@umft.ro (A.L.A.); 3 Department of Oral Implantology, Faculty of Dental Medicine, University of Medicine and Pharmacy “Carol Davila”, 050474 Bucharest, Romania; 4 Multidisciplinary Research Center of Antimicrobial Resistance, Department of Microbiology, Faculty of Medicine, “Victor Babes” University of Medicine and Pharmacy Timisoara, Eftimie Murgu Square 2, 300041 Timisoara, Romania; licker.monica@umft.ro (M.L.); hogea.elena@umft.ro (E.H.); muntean.delia@umft.ro (D.M.); 5 Microbiology Laboratory, “Pius Brinzeu” County Clinical Emergency Hospital, 300723 Timisoara, Romania

**Keywords:** COVID-19, bacterial infections, pneumonia, intensive care unit, antimicrobial resistance

## Abstract

**Background and Objectives:** Secondary bacterial pneumonia can substantially worsen the clinical trajectory of patients hospitalized for Coronavirus Disease 2019 (COVID-19). This study aimed to characterize bacterial superinfections in COVID-19, including pathogen profiles, resistance patterns, inflammatory responses, severity scores, and ICU admission risk. **Methods:** In a retrospective cohort design, we reviewed 141 patients admitted to a single tertiary-care hospital between February 2021 and December 2024. A total of 58 patients had laboratory-confirmed bacterial superinfection by sputum, bronchoalveolar lavage, or blood cultures (superinfection group), whereas 83 had COVID-19 without any documented bacterial pathogens (COVID-only group). We collected detailed microbiological data from sputum, bronchoalveolar lavage (BAL), and blood cultures. Antibiotic sensitivity testing was performed using standard breakpoints for multidrug resistance (MDR). Inflammatory markers (C-reactive protein, procalcitonin, neutrophil-to-lymphocyte ratio, and systemic immune-inflammation index) and the severity indices Acute Physiology and Chronic Health Evaluation (APACHE) II, Confusion, Urea, Respiratory rate, Blood pressure (CURB), and National Early Warning Score (NEWS) were measured at admission. Primary outcomes included intensive care unit (ICU) admission, mechanical ventilation, and mortality. **Results:** Patients in the superinfection group showed significantly elevated inflammatory markers and severity scores compared to the COVID-only group (mean APACHE II of 17.2 vs. 13.8; *p* < 0.001). Pathogens most frequently isolated from sputum and BAL included *Klebsiella pneumoniae* (27.6%) and *Pseudomonas aeruginosa* (20.7%). Multidrug-resistant strains were documented in 32.8% of isolates. The superinfection group had higher ICU admissions (37.9% vs. 19.3%; *p* = 0.01) and more frequent mechanical ventilation (25.9% vs. 9.6%; *p* = 0.01). Mortality trended higher among superinfected patients (15.5% vs. 7.2%; *p* = 0.09). A total of 34% of the cohort had prior antibiotic use, which independently predicted MDR (aOR 2.6, *p* = 0.01). The presence of MDR pathogens such as *Klebsiella pneumoniae* (OR 2.8), *Pseudomonas aeruginosa* (OR 2.5), and *Staphylococcus aureus* (OR 2.1) significantly increases the risk of ICU admission. **Conclusions:** Bacterial superinfection exacerbates inflammation and worsens outcomes in COVID-19 patients, such as a higher risk of ICU admission.

## 1. Introduction

COVID-19, caused by the severe acute respiratory syndrome coronavirus 2 (SARS-CoV-2), predominantly manifests as a respiratory illness with symptoms ranging from mild upper respiratory afflictions to severe pneumonia necessitating mechanical ventilation [1,2,3,4]. While substantial research has focused on the virological dimensions of COVID-19, the impact of secondary bacterial pneumonia on clinical outcomes continues to be critically relevant, particularly against the backdrop of rising antimicrobial resistance and the prevalent overuse of antibiotics [5,6,7]. This issue was especially pronounced during the early stages of the pandemic, when antibiotic prophylaxis for bacterial superinfections was a common approach in various global regions [8,9,10].

Historical data from previous respiratory epidemics, such as those caused by influenza, have shown that bacterial superinfections can significantly increase both morbidity and mortality [11,12]. A similar pattern has emerged during the COVID-19 pandemic, where secondary bacterial infections have been complicating the clinical course for a considerable subset of patients, potentially leading to severe pneumonia, sepsis, and subsequent organ dysfunction [13,14,15,16]. Common bacterial pathogens identified in these instances include Klebsiella pneumoniae, Pseudomonas aeruginosa, and Staphylococcus aureus, which are often linked to ventilator-associated and hospital-acquired pneumonias [17,18,19]. The timely identification of these pathogens through sputum analysis, blood cultures, or bronchoalveolar lavage (BAL) is crucial for guiding effective antibiotic therapy and thereby enhancing outcomes in severe cases [20,21,22].

Furthermore, inflammatory markers such as C-reactive protein (CRP) and procalcitonin (PCT) and ratios like the neutrophil-to-lymphocyte ratio (NLR) and systemic immune-inflammation index (SII) provide valuable insights into the severity of the disease. When used in conjunction with established clinical severity scores like APACHE II (Acute Physiology and Chronic Health Evaluation II), CURB-65 (Confusion, Urea, Respiratory rate, Blood pressure, 65 years of age and older), and NEWS (National Early Warning Score), these biomarkers are instrumental in patient risk stratification and informed decision-making, aiding in the efficient allocation of hospital resources [23,24]. The challenge of managing bacterial superinfections, which can exacerbate respiratory failure and overall systemic compromise, underscores the need for evidence-based strategies to accurately diagnose, isolate, and treat these infections in COVID-19 patients [25]. Moreover, MDR patterns are known to vary by region due to different prevention practices and antibiotic overuse; therefore, another secondary outcome is to understand the local patterns of resistance to antibiotics [26,27]. Achieving a thorough understanding of patient profiles, microbiological patterns, and related inflammatory responses is essential for optimizing treatment protocols and ultimately mitigating the adverse impacts of these infections.

Therefore, this study aims to evaluate the microbiological patterns, inflammatory markers, clinical severity scores, and overall outcomes in COVID-19 patients with and without secondary bacterial pneumonia. By delineating these differences, the aim is to offer insights into enhanced surveillance and tailored antibiotic regimens that could ultimately curb the deleterious effects of bacterial superinfections.

## 2. Materials and Methods

### 2.1. Study Design and Setting

This retrospective cohort study was conducted at a tertiary-care academic hospital in Timisoara, Romania (Eastern Europe), at the Victor Babes Hospital for Infectious Diseases and Pulmonology, a tertiary hospital with approximately 100 beds, serving a population of approximately 50 thousand individuals, affiliated with the Victor Babes University of Medicine and Pharmacy in Timisoara, Romania. The study period ranged from February 2021 to December 2024, encompassing the era during which SARS-CoV-2 variants continued to circulate. Institutional review board approval was obtained prior to data collection. The study protocol was approved by the institutional review board, which follows the Helsinki guidelines for human research, on 28 February 2021 (Ref. 05/2021).

All adult inpatients (≥18 years) with RT-PCR-confirmed COVID-19 were searched for inclusion. The presence of bacterial pneumonia was assessed based on clinical symptoms (fever, increased sputum production), radiologic findings (new focal or multifocal infiltrates on chest imaging), and microbiological confirmation from sputum, BAL, or blood cultures. Patients without evidence of bacterial superinfection were assigned to the COVID-only group.

Inclusion criteria were defined as follows: (1) adults aged 18 years or older; (2) confirmed COVID-19 diagnosis via RT-PCR; (3) admission between January 2021 and December 2024; (4) superinfection group: evidence of bacterial pneumonia through clinical symptoms, radiological findings, and positive microbial cultures from sputum, BAL, or blood; (5) COVID-only group: no signs of bacterial infection, with negative or no growth in all microbial cultures.

Exclusion criteria comprised the following: (1) incomplete medical records; (2) transfers from other facilities lacking initial diagnostic cultures; (3) immunocompromising conditions (cancer, biologic treatments); (4) patients with fungal infections; (5) steroid use for more than two weeks at doses higher than 20mg of prednisone or equivalent.

### 2.2. Patient Selection and Group Allocation

A total of 197 patients with COVID-19 were included by reviewing the medical records. We excluded individuals with incomplete records (n = 14), those transferred from other facilities without initial diagnostic cultures (n = 6), and patients who received palliative care for advanced malignancies irrespective of infection status (n = 36). The remaining 141 patients formed the basis of this analysis. Patients were stratified into two groups based on microbiological evidence of bacterial pneumonia: the superinfection group (n = 58), comprising patients with clinical and radiological signs of pneumonia and at least one positive bacterial culture from sputum, bronchoalveolar lavage (BAL), or blood, and the COVID-only group (n = 83), comprising patients with confirmed COVID-19 but no positive bacterial cultures (all respiratory and blood culture results were negative or showed no growth). Patients were managed according to local COVID-19 treatment protocols, which included supplemental oxygen, antiviral agents (as indicated), corticosteroids, and prophylactic anticoagulation. Antibiotic choices for suspected superinfections were guided by hospital antibiograms, local guidelines, and individual clinical judgment.

Superinfection was defined as secondary bacterial pneumonia that (i) emerged ≥ 48 h after hospital admission for RT-PCR-confirmed COVID-19 and (ii) was supported by (a) new/worsening radiological infiltrates plus ≥ 2 systemic inflammatory signs (fever > 38 °C, CRP > 50 mg L^−1^, or PCT > 0.5 ng mL^−1^), together with (b) bacterial growth ≥ 10⁴ CFU mL^−1^ in BAL or any growth in blood cultures. A ≥ 10⁴ CFU mL^−1^ threshold was used and CPIS ≥ 6 was required for VAP diagnosis.

### 2.3. Data Collection

The microbiological and biological samples in the study were collected within 48 h of admission, as indicated by the initial description of the data collection. This included collecting samples for CRP, procalcitonin, complete blood counts, and electrolytes, alongside microbiological analyses of sputum, BAL fluid, and blood cultures. Bacterial species were first isolated on standard agar plates, followed by routine biochemical panels; identification was confirmed with MALDI-TOF MS.

Antimicrobial susceptibility was determined using the disk diffusion and broth microdilution methods. The criteria for classifying organisms as resistant or susceptible were based on the guidelines provided by the Clinical and Laboratory Standards Institute (CLSI). Additionally, organisms showing resistance to three or more antimicrobial categories were classified as multidrug-resistant, aligning with the established definitions used in clinical microbiology. The detection of ESBLs was performed using the combination disk method, which involves the use of both cephalosporin and cephalosporin combined with clavulanic acid.

Demographic and clinical data included age, sex, body mass index (BMI), relevant comorbidities (e.g., hypertension, chronic obstructive pulmonary disease, chronic kidney disease), and symptom onset. Laboratory measurements obtained within 48 h of admission included CRP, procalcitonin, complete blood counts, and electrolytes. We calculated NLR (absolute neutrophil count ÷ absolute lymphocyte count) and SII ([platelet count × neutrophil count] ÷ lymphocyte count).

Microbiological analyses were performed on sputum samples and, when indicated, on BAL fluid and blood cultures. Organisms were identified using standard laboratory techniques, including culture-based methods and biochemical assays. Acute Physiology and Chronic Health Evaluation (APACHE) II, Confusion, Urea, Respiratory rate, Blood pressure (CURB), and National Early Warning Score (NEWS) scores were recorded at admission. Outcome measures included ICU admission, mechanical ventilation, and in-hospital mortality.

### 2.4. Statistical Analysis

Data were analyzed using SPSS Statistics version 27 (IBM Corp., Armonk, NY, USA). Missing data were addressed using multiple imputation techniques to ensure that the analysis remained robust and representative (5-variable chained equations (20 iterations); concordance between complete cases and imputed results). Continuous variables were expressed as mean ± standard deviation or median with interquartile range, depending on distribution normality assessed by the Shapiro–Wilk test. Between-group comparisons employed Student’s *t*-test or the Mann–Whitney U test. Categorical variables were compared using chi-square or Fisher’s exact test, as appropriate. A *p*-value <0.05 was considered statistically significant. Spearman or Pearson correlation coefficients, as applicable, measured associations between inflammatory markers and severity scores. Subgroup analyses by severity (ICU vs. non-ICU patients) explored differences in bacterial species, inflammatory markers, and outcomes. Risk factor assessment for ICU admission was assessed by a multiple regression analysis with odds ratios (ORs) and confidence intervals (CIs), adjusted by age.

## 3. Results

### 3.1. Patient Demographics

Table 1 outlines key demographic and clinical features. The superinfection group (n = 58) had a marginally higher mean age (64.7 vs. 62.8 years, *p* = 0.31) and a slightly greater proportion of males (56.9% vs. 53.0%, *p* = 0.63), though neither difference reached statistical significance. Similarly, BMI and the prevalence of hypertension, COPD, and chronic kidney disease were comparable in both cohorts (*p* > 0.05). The interval between symptom onset and hospital admission was significantly longer for the superinfection group (6.9 vs. 5.7 days, *p* = 0.005).

The superinfection group revealed distinct pathogenic profiles. *Klebsiella pneumoniae* (27.6%) and *Pseudomonas aeruginosa* (20.7%) were the predominant isolates. *Staphylococcus aureus* was less common (10.3%), yet it still contributed to secondary infections, and *Escherichia coli* was even less common (5.2%). Notably, polymicrobial growth occurred in 8.6% of patients, as presented in Table 2. Of 58 patients, 25.9% (n = 15) had confirmed bacterial pathogens via BAL. *Pseudomonas aeruginosa* (10.3%) and *Klebsiella pneumoniae* (6.9%) were again prominent, mirroring the sputum culture pattern. *Staphylococcus aureus* accounted for 3.4% (n = 2), and polymicrobial infections were detected in 5.2% (n = 3).

### 3.2. Microbiological Analysis

Table 3 captures bloodstream infection rates within the superinfection group. Of 58 patients, 15.5% (n = 9) had positive blood cultures, reflecting either bacterial pneumonia or disseminated infection. Notably, *Klebsiella pneumoniae* and *Staphylococcus aureus* each occurred in 5.2% of the entire group (n = 3 each), while *Pseudomonas aeruginosa* was identified in 3.4% (n = 2). One patient (1.7%) had a polymicrobial bloodstream infection. The majority (84.5%) of superinfected patients had negative blood cultures.

It was observed that *Klebsiella pneumoniae* was the most commonly isolated organism (n = 23), with a multidrug resistance (MDR) rate of 34.8%. Extended-spectrum beta-lactamase (ESBL) and fluoroquinolone resistances were frequently documented. *Pseudomonas aeruginosa* (n = 20) demonstrated a 30.0% MDR rate, with notable resistance against carbapenems and fluoroquinolones. *Staphylococcus aureus* (n = 11) had an MDR rate of 27.3%, largely driven by methicillin-resistant *S. aureus* (MRSA) and, less frequently, macrolide resistance. *Escherichia coli* represented a smaller proportion (n = 3), yet it showed ESBL activity in one isolate. Polymicrobial cultures involved various combinations of these pathogens, yielding an even higher MDR rate of 44.4% (Table 4).

Table 5 compares inflammatory and laboratory measures between the superinfection and COVID-only groups. Consistent with a bacterial component, superinfected patients exhibited higher mean CRP levels (88.5 vs. 71.8 mg/L, *p* < 0.001) and significantly elevated procalcitonin (3.7 vs. 2.0 ng/mL, *p* < 0.001). Leukocytosis was more pronounced in superinfected individuals (10.3 vs. 8.6 × 10^9^/L, *p* = 0.001). The neutrophil-to-lymphocyte ratio (NLR) also showed a clear discrepancy (7.3 vs. 5.4, *p* < 0.001). The systemic immune-inflammation index (SII), which integrates platelets, neutrophils, and lymphocytes, was significantly higher among superinfected patients (1279.6 vs. 993.4 × 10^3^, *p* < 0.001). While platelet counts trended lower in the superinfection group, the difference did not reach statistical significance (*p* = 0.06). However, albumin levels were noticeably reduced (31.9 vs. 34.8 g/L, *p* = 0.001).

### 3.3. Inflammatory Status

**Figure 1:**
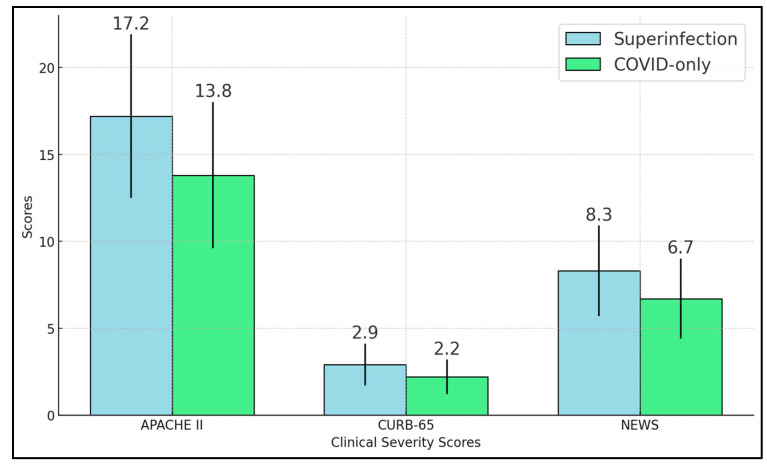
Clinical severity scores.

APACHE II showed a significant elevation in the superinfection group (17.2 vs. 13.8, *p* < 0.001). CURB-65, a pneumonia-specific tool focusing on confusion, blood urea nitrogen, respiratory rate, blood pressure, and age, also revealed significantly higher scores among superinfected individuals (2.9 vs. 2.2, *p* = 0.001). Similarly, the NEWS score was higher in the superinfection cohort (8.3 vs. 6.7, *p* < 0.001), as presented in Figure 1.

Table 6 reviews overall clinical outcomes, demonstrating that superinfected patients were significantly more likely to require ICU admission (37.9% vs. 19.3%, *p* = 0.01) and mechanical ventilation (25.9% vs. 9.6%, *p* = 0.01). While in-hospital mortality trended higher in the superinfection group (15.5% vs. 7.2%, *p* = 0.09), the difference did not meet conventional significance. Hospital stays were notably prolonged for superinfected patients (14.7 vs. 12.2 days, *p* = 0.005). The ICU subgroup analysis reveals that superinfected patients admitted to ICU had higher APACHE II scores (19.0 vs. 15.9, *p* = 0.04) than ICU patients in the COVID-only group. Multidrug-resistant (MDR) organisms were detected in 36.4% of ICU superinfection patients. ICU mortality was higher (27.3% vs. 12.5%) but did not reach statistical significance (*p* = 0.26).

Table 7 presents the results of a multifactorial ANOVA that examines differences between the superinfection (n = 58) and COVID-only (n = 83) groups, and the effect of antibiotic prophylaxis at admission (Yes vs. No). The variables analyzed include C-reactive protein (CRP), procalcitonin, neutrophil-to-lymphocyte ratio (NLR), systemic immune-inflammation index (SII), and APACHE II score. The analysis shows significant group differences in CRP (F = 14.2, *p* < 0.001), procalcitonin (F = 10.8, *p* = 0.001), NLR (F = 11.4, *p* = 0.001), and SII (F = 9.2, *p* = 0.003), with the APACHE II score also being significantly different (F = 15.1, *p* < 0.001). No significant effects were found for antibiotic prophylaxis alone across the variables, with *p*-values ranging from 0.12 to 0.65. Interaction effects between group and prophylaxis were not significant across all variables, with *p*-values ranging from 0.06 to 0.35.

Approximately one-third (31%) of bacterial superinfections involved MDR organisms. Mortality was substantially higher when the infecting pathogen was MDR (33.3 %) versus non-MDR (7.5%). The highest pathogen-specific mortality in the MDR stratum occurred with polymicrobial infections (50%), followed by *S. aureus* (50%) and *K. pneumoniae* (33.3 %). No deaths were recorded among *E. coli* cases, despite one MDR isolate, underscoring pathogen-specific variation in lethality within this cohort (Table 8).

### 3.4. Assessment of Outcomes

**Figure 2:**
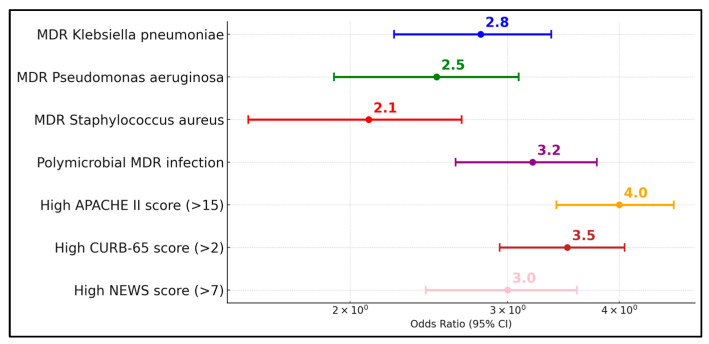
Risk factor assessment for ICU admission.

The data reveal that the presence of MDR pathogens such as *Klebsiella pneumoniae* (odds ratio 2.8, 95% CI 1.6–4.9), *Pseudomonas aeruginosa* (odds ratio 2.5, 95% CI 1.4–4.5), and *Staphylococcus aureus* (odds ratio 2.1, 95% CI 1.2–3.7) significantly increases the risk of ICU admission. Similarly, polymicrobial MDR infections further elevate the risk (odds ratio 3.2, 95% CI 1.8–5.7). Beyond pathogen type, clinical severity scores also present substantial risks: high APACHE II scores (>15) have an odds ratio of 4.0 (95% CI 2.2–7.3), high CURB-65 scores (>2) show an odds ratio of 3.5 (95% CI 2.0–6.1, *p* < 0.001), and high NEWS scores (>7) are associated with an odds ratio of 3.0 (95% CI 1.7–5.4), as presented in Figure 2. A total of 34% of the cohort had prior antibiotic use, which independently predicted MDR (aOR 2.6, *p* = 0.01). Small-cell comparisons with Fisher’s exact test identified the power (β = 0.76 for mortality effect size 0.25).

## 4. Discussion

### 4.1. Analysis of Findings

Our study shows that bacterial superinfections significantly affect the clinical symptoms and microbiological data of hospitalized COVID-19 patients. The superinfection group displayed marked elevations in inflammatory markers, including procalcitonin, neutrophil-to-lymphocyte ratio, and SII, aligning with the anticipated immune response to bacterial pathogens [28,29]. Severity scores—APACHE II, CURB-65, and NEWS—were all significantly higher in these patients, underscoring how secondary infections intensify physiological stress and contribute to more complicated clinical courses [30].

Microbiological data confirm that *Klebsiella pneumoniae* and *Pseudomonas aeruginosa* are principal culprits in these secondary pneumonias, with Staphylococcus aureus also playing a notable role. The detection of multidrug-resistant strains in roughly one-third of isolates further underscores the difficulties in crafting effective empirical antibiotic regimens. These findings suggest that early and repeated respiratory sampling, such as BAL in high-risk cases, is instrumental for targeted therapy. Blood culture positivity, though less common, indicates higher severity and possible systemic spread [31].

Clinically, superinfection strongly correlates with increased ICU admissions, mechanical ventilation, and longer hospital stays [32]. Although mortality differences did not always reach statistical significance, the trend toward worse outcomes is evident. Taken together, the data imply that vigilant monitoring for bacterial superinfection is essential in COVID-19 management. Strategies such as robust antimicrobial stewardship, rapid identification of MDR organisms, and prompt de-escalation of antibiotics based on culture results could help balance the need for broad coverage with the risk of driving resistance.

Other similar studies investigated the prevalence and impact of infections in hospitalized COVID-19 patients. Garcia-Vidal et al. [33] reported a low incidence of community-acquired coinfections at 3.1% and a 4.7% rate of hospital-acquired superinfections among 989 patients, with superinfections mainly due to *Pseudomonas aeruginosa* and *Escherichia coli* occurring approximately 10.6 days post-admission, and an overall mortality of 9.8%. In a similar vein, Scott et al. [34] found a lower rate of bacterial infections at 8% among COVID-19 patients compared to 13% in pre-pandemic pneumonia patients, with most infections being nosocomial, and a higher mortality rate of 15% versus 9% in non-COVID-19 patients. This highlights the critical need for vigilant infection control and management in COVID-19 hospital settings, given the significant impact of nosocomial infections and the heightened mortality associated with such complications.

Yoon et al. [35] identified bacterial superinfections in 30% of the 106 patients studied, with a median onset of 13 days post-ICU admission. This cohort displayed substantially worse outcomes, such as fewer ventilator-free days (median 0 days compared to 19 for those without superinfections) and prolonged ICU (32 days vs. 11 days) and hospital stays (39 days vs. 18 days), although ICU and in-hospital mortality rates did not differ significantly between groups. Similarly, Al-Ali et al. [36] reported superinfections in 17.4% of their 230 patients, occurring approximately 17.6 days after starting antibiotics. These patients experienced longer ICU stays (27.1 days vs. 7.1 days), more complications (92.5% vs. 52.6%), and higher ICU mortality rates (45% vs. 22.1%). Both studies underline the severe challenges posed by superinfections in the management of critically ill COVID-19 patients, emphasizing the need for diligent antibiotic stewardship and monitoring to mitigate these risks and improve patient outcomes.

Søvik et al. [37] found that 43% of their 155 mechanically ventilated patients developed superinfections, predominantly pneumonia, and these were significantly associated with dexamethasone treatment (66% vs. 32%, *p* < 0.0001), autoimmune diseases, and longer ICU stays (26 vs. 17 days, *p* < 0.001). They also noted a higher incidence of invasive fungal infections specifically in patients treated with dexamethasone. Despite these complications, 90-day survival rates did not differ significantly between patients with and without superinfections. In a similar manner, De Francesco et al. [38] reported that 66.3% of the 713 SARS-CoV-2-positive ICU patients developed bacterial and/or fungal superinfections, a significantly higher rate compared to 30% in COVID-19-negative ICU patients. They also identified obesity, length of ICU stay, and duration of mechanical ventilation as risk factors for acquiring superinfections, which were associated with higher mortality rates (45.8% vs. 26.2%, *p* < 0.0001).

Similarly, Moreno-Torres et al. [39] found that 8.5% of 1594 hospitalized patients developed bacterial infections, with urinary tract infections, bacteremia, and pneumonia being the most common types. Independent predictors of these infections included older age, neurological disease, prior immunosuppression, and ICU admission. Although patients with bacterial infections experienced more severe ARDS and had a higher mortality rate (25% vs. 6.7%), bacterial infections themselves were not independently associated with increased mortality when other factors such as age and underlying conditions were considered. In a similar manner, Hughes et al. [40] observed a low incidence of bacterial and fungal coinfections among 836 hospitalized patients during the initial COVID-19 wave in the UK, with only 3.2% showing early confirmed bacterial infections. Their study highlighted that significant bacterial or fungal infections, particularly in the early stages of hospitalization, were uncommon, suggesting that the baseline severity of COVID-19 rather than secondary infections primarily influenced patient outcomes. Both studies underscore the complexity of managing COVID-19 in the hospital setting, pointing to the need for careful consideration of antibiotic use and close monitoring of at-risk patient subgroups.

In their study, Nag and Kaur [41] reported that up to half of COVID-19-related deaths could be linked to superinfections, with ventilator-associated pneumonia (VAP) being the most prevalent, followed by bacteremia and urinary tract infections. In a similar manner, Seitz et al. [42] found that among 117 critically ill COVID-19 patients, 55% developed a superinfection, including a 65.2% rate of VAP, and 13.6% experienced fungal infections (5.9% candidemia and 7.7% COVID-19-associated pulmonary aspergillosis). Both studies underscored that patients with superinfections had prolonged hospital stays and higher mortality, particularly in cases involving multidrug-resistant pathogens or fungal complications. These findings pointed to the urgent need for enhanced surveillance, judicious use of antimicrobials, and further prospective research to guide effective stewardship strategies.

The observed high rates of resistance, including ESBL and fluoroquinolone resistance, necessitate urgent revisions in antimicrobial stewardship programs. Particularly concerning is the correlation between the time from symptom onset to hospital admission and the severity of infection, which suggests that delayed medical intervention may contribute to the advancement of these resistant infections. The high rates of MDR and the presence of ESBLs significantly influence treatment decisions, underscoring the urgent need for enhanced antimicrobial stewardship and targeted therapy. This study’s findings demonstrate a clear association between delayed hospital admission and increased severity of bacterial superinfections, suggesting that earlier intervention could mitigate the progression to severe outcomes.

Recent insights into AMR show an alarming increase in MDR and XDR pathogens, particularly in *Enterobacteriaceae*, *Pseudomonas aeruginosa*, and *Staphylococcus aureus*. Genomic surveillance indicates that resistance genes are rapidly spreading via plasmids, threatening last-resort antibiotics like carbapenems and colistin. The COVID-19 pandemic has worsened AMR due to antibiotic overuse and disrupted monitoring, significantly increasing cases of COVID-19-associated bacterial pneumonia, especially with temperature > 38 °C, LDH > 250 U/L, and d-dimer > 1000 ng/mL [42]. New mechanisms such as biofilm-mediated resistance complicate treatment further. Experts stress the need for rapid diagnostics, phage therapy, and antimicrobial stewardship to combat the growing threat of AMR, projected to cause 10 million deaths annually by 2050 if left unaddressed [43,44].

This study’s findings highlight critical implications for antimicrobial stewardship, underscoring the need for targeted antibiotic therapy based on local antibiogram data to combat the high prevalence of multidrug-resistant organisms like *Klebsiella pneumoniae* and *Pseudomonas aeruginosa*. Rapid diagnostic techniques, such as BAL, are essential for quickly identifying pathogens and tailoring antibiotic treatments, particularly in managing severe and polymicrobial infections. Additionally, the data support rigorous updates to hospital antimicrobial policies and enhanced educational programs for prescribers to optimize antibiotic use, reduce the spread of resistance, and improve patient outcomes in COVID-19 cases complicated by secondary bacterial infections [41].

### 4.2. Study Limitations

Several limitations warrant discussion. First, our study is retrospective and single-center, restricting the generalizability of the findings to other populations with varied microbial landscapes and infection control practices. Second, microbiological testing was clinician-driven, meaning some patients with superinfection might have gone undetected if sampling was not performed. Third, while our cohort size of 141 patients provided meaningful insights, certain subgroup analyses (e.g., ICU outcomes, multidrug resistance patterns) had limited statistical power. Fourth, we did not detail specific treatment regimens, including antivirals, corticosteroids, and antibiotic choices, which may differ between physicians and impact patient trajectories. There is also an explicit limitation by excluding BAL-only diagnoses. Also, the mortality rate was low; therefore, it was not feasible to run a separate analysis for mortality risk. Finally, the observational design prevents causal inferences, although strong associations point to the deleterious effect of secondary bacterial infections in COVID-19. Prospective, multicenter studies with standardized protocols for sampling, antimicrobial therapy, and severity scoring are needed to substantiate these findings and guide best practices.

## 5. Conclusions

Our findings from this single-center cohort indicate that secondary bacterial pneumonia—especially when multidrug-resistant pathogens such as *Klebsiella pneumoniae* or *Pseudomonas aeruginosa* are involved—appears to coincide with higher inflammatory markers and a greater likelihood of ICU admission and mechanical ventilation among hospitalized COVID-19 patients, while the observed increase in mortality did not achieve statistical significance; accordingly, routine, rapid microbiological work-ups may assist in refining antibiotic choices and potentially improving outcomes, yet larger prospective and multicenter studies are needed before firm clinical recommendations can be made.

## Figures and Tables

**Table 1:** Baseline demographics and clinical characteristics.

Variable	Superinfection (n = 58)	COVID-Only (n = 83)	*p*-Value
Age (years)	64.7 ± 11.3	62.8 ± 10.1	0.31
Male (%)	56.9 (33/58)	53.0 (44/83)	0.63
BMI (kg/m^2^)	28.2 ± 5.1	27.5 ± 4.8	0.38
Hypertension (%)	63.8 (37/58)	57.8 (48/83)	0.47
COPD (%)	20.7 (12/58)	14.5 (12/83)	0.32
Chronic Kidney Disease (%)	15.5 (9/58)	12.0 (10/83)	0.52
Days from Symptom Onset to Admission	6.9 ± 2.2	5.7 ± 1.8	0.005

**Table 2:** Sputum and BAL cultures in COVID-19 patients with superinfection.

Sputum Organism	Positive Sputum Cultures (n = 58)	BAL Organism	Positive BAL Cultures (n = 15)
*Klebsiella pneumoniae* (%)	27.6 (16/58)	*Pseudomonas aeruginosa* (%)	10.4 (6/58)
*Streptococcus* spp. (%)	27.6 (16/58)	*Klebsiella pneumoniae* (%)	6.9 (4/58)
*Pseudomonas aeruginosa* (%)	20.7 (12/58)	*Staphylococcus aureus* (%)	3.4 (2/58)
*Staphylococcus aureus* (%)	10.3 (6/58)	*Streptococcus* spp. (%)	3.4 (2/58)
*Escherichia coli* (%)	5.2 (3/58)	Polymicrobial (%)	5.2 (3/58)
Polymicrobial Growth (%)	8.6 (5/58)	Negative BAL/Not Performed	74.1 (43/58)

**Table 3:** Blood culture results in superinfection group.

Blood Cultures	Frequency (n = 58)	Percentage (%)
Positive Blood Cultures	9	15.5
**Isolated Organisms**		
*Klebsiella pneumoniae*	3	5.2
*Staphylococcus aureus*	3	5.2
*Pseudomonas aeruginosa*	2	3.4
Polymicrobial Mixtures	1	1.7
Negative Blood Cultures	49	84.5

**Table 4:** Antibiotic resistance patterns and multidrug resistance (MDR).

Organism	Total Isolates	MDR Rate (%)	Common Resistant Classes
*Klebsiella pneumoniae*	23	34.8 (8/23)	β-lactams (ESBLs), fluoroquinolones
*Pseudomonas aeruginosa*	20	30.0 (6/20)	Carbapenems, fluoroquinolones
*Staphylococcus aureus*	11	27.3 (3/11)	Methicillin (MRSA), macrolides
Polymicrobial (mixed)	9	44.4 (4/9)	Varies by combination
*Escherichia coli*	3	33.3 (1/3)	β-lactams (ESBLs)

**Table 5:** Inflammatory and laboratory parameters compared between patients with bacterial superinfection and COVID-only patients.

Parameter	Superinfection (n = 58)	COVID-Only (n = 83)	*p*-Value
CRP (mg/L)	88.5 ± 25.7	71.8 ± 22.6	<0.001
Procalcitonin (ng/mL)	3.7 ± 1.1	2.0 ± 0.8	<0.001
WBC (×10^9^/L)	10.3 ± 3.1	8.6 ± 2.7	0.001
NLR	7.3 ± 2.6	5.4 ± 2.0	<0.001
SII (×10^3^)	1279.6 ± 393.2	993.4 ± 331.1	<0.001
Platelets (×10^9^/L)	211.2 ± 65.5	229.1 ± 69.7	0.06
Albumin (g/L)	31.9 ± 5.5	34.8 ± 5.3	0.001

**Table 6:** Clinical outcomes and subgroup analysis in COVID-19 patients with and without bacterial superinfection.

Outcome	Superinfection (n = 58)	COVID-Only (n = 83)	*p*-Value
ICU Admission (%)	37.9 (22/58)	19.3 (16/83)	0.01
Mechanical Ventilation (%)	25.9 (15/58)	9.6 (8/83)	0.01
In-Hospital Mortality (%)	15.5 (9/58)	7.2 (6/83)	0.09
Length of Stay (days)	14.7 ± 5.1	12.2 ± 4.4	0.005
**ICU Subgroup**			0.11
APACHE II in ICU patients	19.0 ± 5.2 (n = 22)	15.9 ± 4.7 (n = 16)	0.04
MDR Organisms in ICU (%)	36.4 (8/22)	N/A	N/A
ICU Mortality (%)	27.3 (6/22)	12.5 (2/16)	0.26

**Table 7:** Multifactor ANOVA results differentiating superinfection group from COVID-only group.

Variable	F (Group)	*p* (Group)	F (Proph)	*p* (Proph)	F (Interaction)	*p* (Interaction)
CRP (mg/L)	14.2	<0.001	0.75	0.39	2.11	0.15
Procalcitonin (ng/mL)	10.8	0.001	1.9	0.17	3.65	0.06
NLR (neutrophil-to-lymphocyte ratio)	11.4	0.001	2.45	0.12	1.1	0.29
SII (×10^3^)	9.2	0.003	0.55	0.46	2.29	0.13
APACHE II (score)	15.1	<0.001	0.21	0.65	0.88	0.35

Group: superinfection (n = 58) vs. COVID-only (n = 83); Proph: antibiotic prophylaxis (Yes vs. No) at admission; CRP: C-reactive protein; NLR: neutrophil-to-lymphocyte ratio; SII: systemic immune-inflammation index.

**Table 8:** In-hospital mortality stratified by pathogen and multidrug resistance (MDR) status (superinfection cohort, n = 58).

Pathogen	Total Cases, n	MDR Cases, n (%) †	Mortality in Non-MDR Isolates, n (%) ‡	Mortality in MDR Isolates, n (%) ‡
*Klebsiella pneumoniae*	16	6 (37.5)	1 (10.0)	2 (33.3)
*Pseudomonas aeruginosa*	12	4 (33.3)	1 (12.5)	1 (25.0)
*Staphylococcus aureus*	6	2 (33.3)	0 (0.0)	1 (50.0)
*Streptococcus* spp.	16	1 (6.2)	1 (6.7)	0 (0.0)
*Escherichia coli*	3	1 (33.3)	0 (0.0)	0 (0.0)
Polymicrobial cultures §	5	4 (80.0)	0 (0.0)	2 (50.0)
**All pathogens**	**58**	**18 (31.0)**	**3 (7.5)**	**6 (33.3)**

† MDR defined as resistance to ≥3 antimicrobial classes on susceptibility testing. ‡ Percentages are calculated within the respective subgroup (e.g., deaths ÷ non-MDR cases for “Mortality in non-MDR isolates”). § Polymicrobial episodes counted when ≥2 bacterial species were isolated from the same respiratory or blood culture; MDR status reflects the presence of any MDR organism within the mixture.

## Data Availability

The data presented in this study are available on request from the corresponding author.
